# Use of Antidiabetic drugs during pregnancy among U.S. women with Livebirth deliveries in the Mini-Sentinel system

**DOI:** 10.1186/s12884-019-2609-8

**Published:** 2019-11-27

**Authors:** Katrina Mott, Marsha E. Reichman, Sengwee Toh, Caren Kieswetter, Katherine Haffenreffer, Susan E. Andrade

**Affiliations:** 1 Department of Epidemiology, Harvard T.H. Chan School of Public Health, 677 Huntington Ave, Boston, MA USA; 2 Center for Drug Evaluation and Research, U.S. Food and Drug Administration, Silver Spring, MD USA; 3 Department of Population Medicine, Harvard Medical School and Harvard Pilgrim Health Care Institute, Boston, MA USA; 4 Meyers Primary Care Institute (Fallon Community Health Plan, Reliant Medical Group, and University of Massachusetts Medical School), Worcester, MA USA

**Keywords:** Pregnancy, Glyburide, Insulin, Gestational diabetes

## Abstract

**Background:**

As the prevalence of diabetes mellitus increases in the population, the exposure to antidiabetic drugs (ADDs) during pregnancies is expected to grow, as has been seen over the last decade. The objective of this study was to estimate the prevalence of ADD use during pregnancy among women in the Mini-Sentinel Distributed Database (MSDD) who delivered a liveborn infant.

**Methods:**

We identified qualifying livebirth pregnancies among women aged 10 to 54 years in the MSDD from 2001 to 2013. ADD use was estimated using outpatient pharmacy dispensing claims and days-supplied among three cohorts: all livebirth pregnancies, pregnancies among women with pre-existing diabetes, and pregnancies among women without prior ADD use.

**Results:**

Among the 1.9 million pregnancies in the MSDD that resulted in a livebirth from 2001 to 2013, 4.4% were exposed to an ADD. Of the 15,606 pregnancies (0.8%) with pre-existing diabetes, 92.8% were also exposed during the pregnancy period. The most commonly used product in these pregnancies was insulin (75.6% of pregnancies). In contrast, in pregnancies of women without prior ADD use, the most commonly used products were glyburide and insulin, and most of these users were diagnosed with gestational diabetes.

**Conclusions:**

Patterns of ADD use during pregnancy described here, along with changes in disease incidence and management, highlight the importance of continuing surveillance of ADD utilization patterns and examining the safety and effectiveness of these products in pregnancy.

## Key points

1. From 2001 to 2013, 4.4% (*n* = 82,676) of live birth pregnancies in the database were exposed to an antidiabetic drug (ADD).
2. Over the gestational period, increases in ADD use are mostly due to increases in use of two agents: glyburide and insulin.
3. Of pregnancies among women with pre-existing diabetes, 93% were dispensed an ADD during the gestational period.
4. In pregnancies among women without prior ADD use, insulin and sulfonylureas were the most commonly used agents.

## Introduction

There is a need for ongoing routine surveillance of medication use during pregnancy, as new drugs become available and prescribing trends and recommendations change. Up to 9% of pregnant women have pre-existing diabetes mellitus or develop gestational diabetes, which may require drug therapy [1–6]. The prevalence of gestational diabetes has been increasing over the last two decades [1–6]. Unmanaged diabetes in pregnancy, leading to hyperglycemia, is associated with excess risk of macrosomia, stillbirths, and other neonatal complications [7].

The American Congress of Obstetricians and Gynecologists (ACOG) Practice Bulletin #30 in 2001 recommended adding insulin if glycemic control could not be achieved with nutritional therapy alone for women with gestational diabetes [8]. Several randomized controlled trials and observational studies, which compared glyburide to insulin for gestational diabetes, showed the two therapies to be comparable in achieving glycemic control [9]. In 2013, the ACOG guidelines were revised (Practice Bulletin #137) to state that insulin and oral ADDs (e.g., glyburide and metformin) are equally efficacious, and could be considered for first-line therapy [9]. The use of glyburide increased during this time period [9]. The American Diabetes Association guidelines (2015) lists insulin and metformin as preferred treatments, and states that glyburide may be used, but may have a higher rate of neonatal hypoglycemia and macrosomia [10].

For women with pre-existing diabetes who become pregnant, an ACOG Practice Bulletin (#60) in 2005 and a consensus statement by the American Diabetes Association in 2008 recommend insulin as the primary method of glycemic control [7, 11]. The ACOG guideline recommended stopping oral ADDs and switching to insulin as early in the pregnancy as possible [11].

The goal of this paper is to describe patterns of ADD use during pregnancy in the large cohort of livebirth pregnancies in the Mini-Sentinel Distributed Database (MSDD) from 2001 to 2013.

## Methods

### Data source

The U.S. Food and Drug Administration (FDA) Mini-Sentinel pilot project was a collaboration to build an active surveillance system for medical product safety [12]. This pilot project, which has now transitioned to the full-fledged Sentinel system, built a distributed database of insurance claims and administrative data using a common data model to create the MSDD. As of July 2014, the database included medical claims with procedure and diagnosis codes, pharmacy claims, and enrollment and demographic information for more than 178 million individuals. For this project, 15 data partners contributed to the analysis. The data partners performed standardized data analysis using an analytic tool developed by the Mini-Sentinel Operations Center and returned the summary-level results to the Operations Center for final aggregation and analysis. Data partners contributed data for varying time periods throughout the study period, with the majority of included pregnancies occurring after 2009. This project was conducted under FDA’s public health authority and was not under the purview of the Institutional Review Board.

### Analytic tool

This analysis utilized the Mini-Sentinel pregnancy analytic tool to identify women aged 10 to 54 years who delivered a liveborn infant between 2001 and 2013 [13]. It identified maternal characteristics of the eligible pregnancies, including year of delivery, maternal age, pre/post-term birth, and pre-existing diabetes. To be included, women had to be enrolled in a health plan offered by an MSDD data partner which included medical and pharmacy benefits for at least 480 days prior to delivery. A woman could contribute multiple pregnancies if she met these criteria. Pregnancy periods, including trimesters, were calculated using a validated algorithm developed in the Medication Use in Pregnancy Risk Evaluation Program (MEPREP) and Post-Licensure Rapid Immunization Safety Monitoring (PRISM) projects to estimate gestational age at birth [14–16].

After cohort identification, the analytic tool determined medication exposure based on outpatient pharmacy dispensing claims for ADDs, identified by National Drug Code, during and prior to the pregnancy period. Exposure was defined using both dispense dates and days-supplied information in the MSDD. First trimester was defined as days 0 to 90 (with day 0 calculated as the estimated start of the pregnancy), the second trimester as days 91 to 180, and the third trimester as day 181 through the admit date of the hospital admission for delivery. All drug dispensing events were counted, so that a pregnancy could be counted in multiple drug exposure categories.

### Cohorts

For this analysis, three cohorts were identified. First, the cohort of all eligible livebirth pregnancies was defined using the criteria described above to capture any qualifying livebirth pregnancy during the time period. Second, the cohort of pregnancies among women with pre-existing diabetes was identified as any pregnancy for which the mother had a dispensing of a non-metformin ADD or a dispensing of metformin with a diabetes diagnosis code (ICD-9-CM 250.x) at any time in the 183 days prior to the start of pregnancy. We excluded metformin use that lacked a diabetes diagnosis code because metformin is also used to treat non-diabetic conditions, such as polycystic ovary syndrome. The third cohort consisted of pregnancies in women without any ADD dispensing in the 183 days prior to the start of pregnancy. To estimate the trends of ADD use for gestational diabetes, we identified pregnancies in the third cohort as women who were dispensed an ADD during the pregnancy period, and who met the following definition of gestational diabetes: a diagnosis code for gestational diabetes (ICD-9-CM 648.8) in the 2nd or 3rd trimester and no prior diabetes mellitus diagnosis.

### Statistical analysis

In the three cohorts of pregnancies, we identified ADD use and specific agent use any time during pregnancy and by trimester. The percent of pregnancies with use of each product are presented to compare use across products, and through the pregnancy trimesters. We also stratified use by age group to identify differences in use patterns by maternal age. Prevalence ratios (PR) were calculated for the primary findings.

## Results

The *cohort of all livebirth pregnancies* consisted of 1,895,604 live birth pregnancies in 1.6 million women between 2001 and 2013, identified with the pregnancy algorithm in the MSDD (Table 1). For most pregnancies (58.3%), women were between ages 25 to 34 at the time of delivery. Approximately 7.7% of deliveries had a code for preterm birth, while 13.7% had a code for post-term birth. Of the total cohort, 4.4% (*n* = 82,676) of pregnancies had exposure to an ADD. Table 2 displays the use of ADD products by trimester in the total pregnancy cohort. Use of any ADD increased from the 1st to 3rd trimester (2.1 to 3.4% of pregnancies, prevalence ratio = 1.59, 95%CI (1,57, 1.61)). This increase was due primarily to an increase in use of glyburide (0.1 to 1.4%, PR = 13.91, 95%CI (13.27, 14.57)) and increase in use of insulin (0.7 to 1.8%, PR = 2.72, 95%CI (2.67, 2.78)) over the course of the pregnancy period. In the 90 days before pregnancy, 1.6% of pregnancies had exposure to metformin, but use of metformin decreased to 0.5% by the 3rd trimester. As expected, exposure to ADDs increased with increasing maternal age (Table 3). The trend was similar for the three drug categories with the most use (metformin, sulfonylureas, and insulin).

**Table 1:** Characteristics of the cohort of all livebirth pregnancies and the cohort of pregnancies among women with pre-existing diabetes between 2001 and 2013 in the MSDD

Characteristic		Cohort of all livebirth pregnancies	Cohort of pregnancies in women with pre-existing diabetes
Total unique women with a pregnancy episode		1,598,705	14,216
Total unique pregnancies		1,895,604 (100.00%)	15,606 (100.00%)
Total unique pregnancies with ADD		82,676 (4.4%)	14,488 (92.8%)
Total unique pregnancies without ADD		1,812,928 (95.6%)	1118 (7.2%)
Maternal age at delivery, years	< 20 20–24 25–29 30–34 35–39 40–44 45–54	115,584 (6.1%) 260,013 (13.7%) 495,250 (26.1%) 610,703 (32.2%) 329,080 (17.4%) 76,995 (4.1%) 7979 (0.4%)	404 (2.6%) 1608 (10.3%) 3545 (22.7%) 4949 (31.7%) 3745 (24.0%) 1082 (6.9%) 273 (1.8%)
Year of delivery	2001 2002 2003 2004 2005 2006 2007 2008 2009 2010 2011 2012 2013	41,870 (2.21%) 62,345 (3.29%) 62,310 (3.29%) 61,544 (3.25%) 64,573 (3.41%) 68,056 (3.59%) 122,674 (6.47%) 146,443 (7.73%) 246,203 (12.99%) 275,835 (14.55%) 263,694 (13.91%) 252,272 (13.31%) 227,785 (12.02%)	245 (1.57%) 4.62 (2.96%) 492 (3.15%) 585 (3.75%) 581 (3.72%) 626 (4.01%) 1040 (6.66%) 1271 (8.14%) 1946 (12.47%) 2330 (14.93%) 2132 (13.66%) 2107 (13.50%) 1789 (11.46%)
Any code for preterm birth		146,523 (7.7%)	3108 (19.9%)
Any code for postterm birth		259,572 (13.7%)	309 (2.0%)

**Table 2:** Antidiabetic Drug use, by trimester, in the cohort of all livebirth pregnancies during 2001–2013 in the MSDD

	Use in the 90 Days Before Pregnancy	Any Use During Pregnancy	Any Use, First Trimester	Any Use, Second Trimester	Any Use, Third Trimester
Total unique pregnancies	1,895,604 (100%)	1,895,604 (100%)	1,895,604 (100%)	1,895,604 (100%)	1,895,122 (100%)
Drug Class or Product					
Use of Any Drug	38,017 (2.0%)	82,676 (4.4%)	40,013 (2.1%)	35,587 (1.9%)	63,483 (3.4%)
Alpha-Glucosidase Inhibitors	21 (<0.1%)	149 (<0.1%)	19 (< 0.1%)	24 (<0.1%)	129 (<0.1%)
Amylin Analog	55 (<0.1%)	48 (<0.1%)	46 (<0.1%)	9 (< 0.1%)	5 (< 0.1%)
Metformin	30,194 (1.6%)	32,757 (1.7%)	29,431 (1.6%)	15,827 (0.8%)	9897 (0.5%)
Dipeptidyl Peptidase-4 Inhibitors	226 (< 0.1%)	242 (< 0.1%)	227 (< 0.1%)	87 (< 0.1%)	50 (< 0.1%)
Glucagon-like Peptide-1 Agonists	403 (<0.1%)	333 (<0.1%)	316 (<0.1%)	77 (<0.1%)	32 (<0.1%)
Meglitinide Analogs	45 (<0.1%)	44 (<0.1%)	43 (<0.1%)	12 (<0.1%)	2 (<0.1%)
Sulfonylureas – Use of Any Drug	2018 (0.1%)	28,240 (1.5%)	2748 (0.1%)	6325 (0.3%)	26,234 (1.4%)
Glimepiride	312 (<0.1%)	280 (<0.1%)	255 (<0.1%)	97 (<0.1%)	61 (<0.1%)
Glipizide	675 (<0.1%)	768 (< 0.1%)	617 (<0.1%)	316 (<0.1%)	218 (<0.1%)
Glyburide	976 (0.1%)	27,269 (1.4%)	1868 (0.1%)	5905 (0.3%)	25,973 (1.4%)
Tolazamide	80 (<0.1%)	82 (<0.1%)	82 (<0.1%)	53 (<0.1%)	23 (<0.1%)
Thiazolidinediones	965 (0.1%)	874 (0.1%)	841 (<0.1%)	274 (<0.1%)	123 (<0.1%)
Combination Products	565 (<0.1%)	633 (<0.1%)	520 (<0.1%)	204 (<0.1%)	155 (<0.1%)
Insulin – Any Injectable Insulin	7351 (0.4%)	34,476 (1.8%)	12,289 (0.7%)	18,028 (0.9%)	33,447 (1.8%)
Rapid-acting	5842 (0.3%)	25,959 (1.4%)	10,074 (0.5%)	14,602 (0.8%)	24,705 (1.3%)
Intermediate-acting	1803 (0.1%)	26,103 (1.4%)	6607 (0.4%)	11,824 (0.6%)	24,745 (1.3%)
Long-acting	2394 (0.1%)	4834 (0.3%)	2846 (0.2%)	2791 (0.2%)	3436 (0.2%)
Other-acting	598 (<0.1%)	1556 (0.1%)	854 (0.1%)	794 (0.1%)	976 (0.1%)

**Table 3:** Antidiabetic Drug use, by maternal age, in all livebirth pregnancies during 2001–2013 in the MSDD

	< 20 years	20–24 years	25–29 years	30–34 years	35–39 years	40–44 years	45–54 years
Total unique pregnancies	115,584 (100%)	260,013 (100%)	495,250 (100%)	610,703 (100%)	329,080 (100%)	76,995 (100%)	7979 (100%)
Drug product/Class							
Any Antidiabetic Drug	1004 (0.87%)	5227 (2.01%)	18,775 (3.79%)	30,327 (4.97%)	20,788 (6.32%)	5874 (7.63%)	681 (8.53%)
Alpha-Glucosidase Inhibitors	4 (0.00%)	10 (0.00%)	31 (0.01%)	54 (0.01%)	30 (0.01%)	18 (0.02%)	2 (0.03%)
Amylin Analog	1 (0.00%)	4 (0.00%)	15 (0.00%)	19 (0.00%)	6 (0.00%)	3 (0.00%)	0 (0.00%)
Metformin	294 (0.25%)	1839 (0.71%)	8616 (1.74%)	12,809 (2.10%)	7238 (2.20%)	1687 (2.19%)	274 (3.43%)
Dipeptidyl Peptidase-4 Inhibitors	1 (0.00%)	4 (0.00%)	34 (0.01%)	69 (0.01%)	83 (0.03%)	30 (0.04%)	21 (0.26%)
Glucagon-like Peptide-1 Receptor Agonists	4 (0.00%)	12 (0.00%)	58 (0.01%)	109 (0.02%)	98 (0.03%)	36 (0.05%)	16 (0.20%)
Meglitinide Analogs	1 (0.00%)	8 (0.00%)	8 (0.00%)	13 (0.00%)	9 (0.00%)	3 (0.00%)	2 (0.03%)
Sulfonylureas	237 (0.21%)	1452 (0.56%)	5542 (1.12%)	10,151 (1.66%)	8012 (2.43%)	2570 (3.34%)	276 (3.46%)
Thiazolidinediones	17 (0.01%)	69 (0.03%)	208 (0.04%)	268 (0.04%)	196 (0.06%)	73 (0.09%)	43 (0.54%)
Combination Products	10 (0.01%)	55 (0.02%)	117 (0.02%)	197 (0.03%)	177 (0.05%)	52 (0.07%)	25 (0.31%)
Insulin	590 (0.51%)	2562 (0.99%)	6986 (1.41%)	11,868 (1.94%)	9392 (2.85%)	2813 (3.65%)	265 (3.32%)

**Fig. 1:**
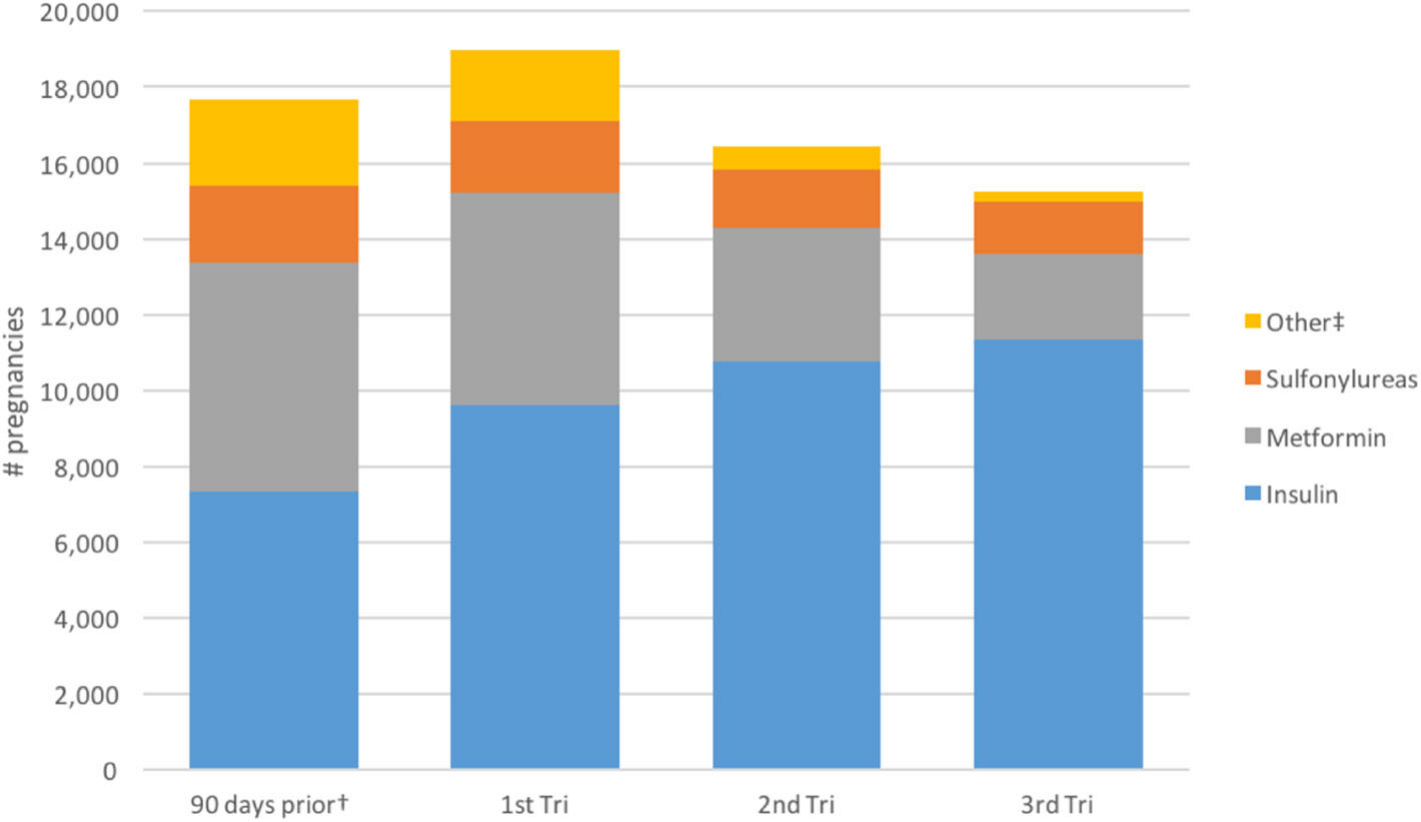
Antidiabetic drug use, by trimester, in the cohort of pregnancies among women with pre-existing diabetes during 2001–2013 in the MSDD. † 90 days prior is the period of 90 days prior to the calculated start of pregnancy based on the pregnancy period algorithm using livebirth delivery claim codes. 1st Tri, 2nd Tri, and 3rd Tri refer to the gestational trimesters, also calculated using the pregnancy period algorithm based on delivery codes. Individual pregnancies could be counted in multiple gestational terms and for multiple drug categories. ‡Other category includes alpha-glucosidase inhibitors, meglitinide analogs, amylin analog, DPP-4 inhibitors, GLP-1 receptor agonists, thiazolidinediones, and combination products

There were 15,606 *pregnancies among women with pre-existing diabetes*, of which 92.8% had an ADD exposure during the pregnancy (Table 4). Compared to all livebirth pregnancies, pregnancies with pre-existing diabetes were more likely to be among older women (32.7% of the pre-existing diabetes cohort vs. 21.9% of the total cohort was over age 35), and were more likely to have a pre-term birth (19.9% vs. 7.7%) (Table 1). In this cohort of women with pre-existing diabetes, the prevalence of ADD use remained steady over the course of pregnancy (85.4, 82.5, and 83.4% in each successive trimester, respectively), as shown in Table 4. The most commonly used product during pregnancy was insulin (75.6% of pregnancies), followed by metformin as the second most commonly used product (37.7%) (PR = 2.00, 95%CI (1.96, 2.05), Fig. 1). Metformin use among these pregnancies decreased over the course of pregnancy, from 35.7% in the first trimester to 14.5% in the third trimester (Table 4, Fig. 1). Sulfonylureas were also commonly used: glyburide at any time in 12.8% of pregnancies (*n* = 2001) and consistently across trimesters (7.0% of pregnancies in the 1st trimester to 8.1% in the 3rd), and glipizide at any time in 3.8% of pregnancies. Notably, pre-term delivery was more common among women with pre-existing diabetes (19.9% vs. 7.7% in total cohort), so the third trimesters would, on average, be shorter than the second and first trimesters -- effectively having fewer days on which a prescription could be filled. There are large differences in the distribution of drugs used by maternal age group in this cohort (Table 5). The proportion of pregnancies exposed to metformin was more than twice as high in women over age 40 compared to the proportion in women under age 25 (PR = 2.82, 95%CI (2.56, 3.13)). Similarly, the proportion of pregnancies exposed to a sulfonylurea was 30% over age 45, compared to less than 7% in pregnancies in women under age 20. Between 70 and 80% of pregnancies in women under age 45 with pre-existing diabetes were exposed to insulin.

**Table 4:** Antidiabetic drug use, by trimester, in livebirth pregnancies among women with pre-existing diabetes during 2001–2013 in the MSDD

	Use in the 90 Days Before Pregnancy	Any Use During Pregnancy	Any Use, First Trimester	Any Use, Second Trimester	Any Use, Third Trimester
Total unique pregnancies	15,606 (100%)	15,606 (100%)	15,606 (100%)	15,606 (100%)	15,594 (100%)
Drug Product/Class					
Use of Any Drug	13,847 (88.7%)	14,488 (92.8%)	13,322 (85.4%)	12,876 (82.5%)	12,998 (83.4%)
Metformin	6024 (38.6%)	5886 (37.7%)	5572 (35.7%)	3533 (22.6%)	2257 (14.5%)
Sulfonylureas – Any Drug	2018 (12.9%)	2803 (18.0%)	1916 (12.3%)	1532 (9.8%)	1414 (9.1%)
*Glipizide*	675 (4.3%)	596 (3.8%)	573 (3.7%)	270 (1.7%)	91 (0.6%)
*Glyburide*	976 (6.3%)	2001 (12.8%)	1097 (7.0%)	1162 (7.5%)	1268 (8.1%)
Thiazolidinediones	965 (6.2%)	800 (5.1%)	786 (5.0%)	258 (1.7%)	104 (0.7%)
Combination Products	565 (3.6%)	502 (3.2%)	480 (3.1%)	166 (1.1%)	73 (0.5%)
Insulin	7351 (47.1%)	11,796 (75.6%)	9625 (61.7%)	10,776 (69.1%)	11,328 (72.6%)

**Table 5:** Antidiabetic drug use, by maternal age, in livebirth pregnancies among women with pre-existing diabetes during 2001–2013 in the MSDD

	< 20 years	20–24 years	25–29 years	30–34 years	35–39 years	40–44 years	45–54 years
Total unique pregnancies	404 (100%)	1608 (100%)	3545 (100%)	4949 (100%)	3745 (100%)	1082 (100%)	273 (100%)
Drug product/Class							
Any Antidiabetic Drug	365 (90.35%)	1419 (88.25%)	3262 (92.02%)	4635 (93.66%)	3536 (94.42%)	1010 (93.35%)	261 (95.60%)
Alpha-Glucosidase Inhibitors	0 (0.00%)	2 (0.12%)	8 (0.23%)	9 (0.18%)	3 (0.08%)	4 (0.37%)	2 (0.73%)
Amylin Analog	1 (0.25%)	3 (0.19%)	14 (0.39%)	17 (0.34%)	6 (0.16%)	3 (0.28%)	0 (0.00%)
Metformin	87 (21.53%)	311 (19.34%)	1102 (31.09%)	1906 (38.51%)	1722 (45.98%)	598 (55.27%)	160 (58.61%)
Dipeptidyl Peptidase-4 Inhibitors	1 (0.25%)	3 (0.19%)	32 (0.90%)	66 (1.33%)	75 (2.00%)	27 (2.50%)	19 (6.96%)
Glucagon-like Peptide-1 Receptor Agonists	3 (0.74%)	11 (0.68%)	56 (1.58%)	103 (2.08%)	94 (2.51%)	34 (3.14%)	15 (5.49%)
Meglitinide Analogs	1 (0.25%)	7 (0.44%)	8 (0.23%)	13 (0.26%)	9 (0.24%)	3 (0.28%)	2 (0.73%)
Sulfonylureas	28 (6.93%)	184 (11.44%)	512 (14.44%)	907 (18.33%)	833 (22.24%)	255 (23.57%)	84 (30.77%)
Thiazolidinediones	14 (3.47%)	64 (3.98%)	181 (5.11%)	246 (4.97%)	187 (4.99%)	68 (6.28%)	40 (14.65%)
Combination Products	6 (1.49%)	37 (2.30%)	84 (2.37%)	155 (3.13%)	155 (4.14%)	43 (3.97%)	22 (8.06%)
Insulin	321 (79.46%)	1237 (76.93%)	2701 (76.19%)	3767 (76.12%)	2868 (76.58%)	789 (72.92%)	113 (41.39%)

**Fig. 2:**
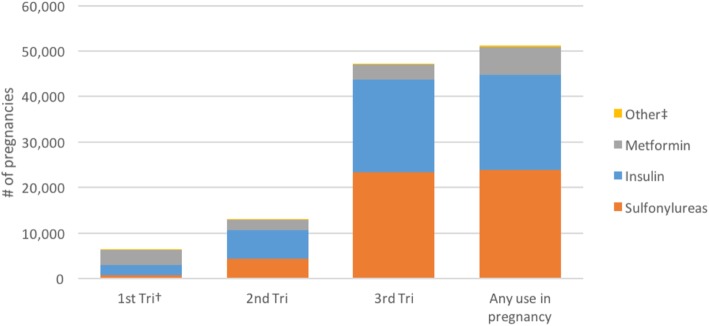
Antidiabetic drug use, by trimester, in the cohort of pregnancies among women with no prior ADD use during 2001–2013 in the MSDD. † 1st Tri, 2nd Tri, and 3rd Tri refer to the three gestational trimesters, calculated using the pregnancy period algorithm based on delivery codes. Individual pregnancies could be counted in multiple gestational terms and for multiple drug categories. ‡ Other category includes alpha-glucosidase inhibitors, meglitinide analogs, amylin analog, DPP-4 inhibitors, GLP-1 receptor agonists, thiazolidinediones, and combination products

Among *pregnancies without prior use of any ADD* in the 183 days before last menstrual period (LMP), the most commonly used ADDs in pregnancy were sulfonylureas, followed by insulin and metformin (Fig. 2). In this cohort, as expected for gestational diabetes, exposure to ADDs increased over the course of pregnancy with most use occurring in the 3rd trimester when the diagnosis is made (reflected by the use of sulfonylureas and insulin). However, a proportion of pregnancies without prior use of any ADD did not meet both criteria of our gestational diabetes algorithm definition: a diagnosis code for gestational diabetes in the 2nd or 3rd trimester *and* no prior diabetes mellitus diagnosis (5.8% of sulfonylurea-exposed pregnancies, 14.4% of insulin-exposed pregnancies, and 50.2% of metformin-exposed pregnancies).

## Discussion

In the MSDD cohort of livebirth pregnancies from 2001 to 2013, 4.4% were exposed to an ADD during pregnancy. Use of insulin and glyburide increased in prevalence over the pregnancy period, which is likely due to a combination of newly diagnosed gestational diabetes and a switch to these treatments from other oral drugs among women with pre-existing diabetes. In pregnancies among the women who might have gestational diabetes based on their ADD use pattern (i.e. no ADD use prior to pregnancy with ADD initiation during pregnancy), not all of the women met our gestational diabetes definition based on the algorithm described above. We suspect two possible explanations account for the women not meeting the definition: 1) they had untreated diabetes mellitus prior to pregnancy and were started on ADD therapy during pregnancy. Therefore, the algorithm appropriately excluded these women from being assigned a gestational diabetes indicator; or 2) they were clinically diagnosed and treated for gestational diabetes but a claim with the gestational diabetes diagnosis code was not filed with their insurance. Errors in coding or claims may also be responsible for some proportion of this discrepancy.

Many of the findings in this analysis were similar to those of previous work, particularly in the MEPREP database [17]. Specifically, the proportion of pregnancies with metformin, glyburide, and insulin exposure were comparable in the two studies. This similarity is expected, since 8 out of the 15 data partners in this analysis also participated in MEPREP. In the cohort of women with pre-existing diabetes in this study, at least 83% of pregnancies were exposed to an ADD in the second or third trimester, in contrast to the 56.5% reported by MEPREP although different definitions of prior ADD exposure were used. The MEPREP study included ADD use within 120 days prior to pregnancy and may have included women using metformin for reasons other than glycemic control who would not be expected to continue use during pregnancy [17].

The major strengths of this study are the size of the pregnancy cohort and the ability to define sub-cohorts based on prior diagnoses or prescriptions dispensed. The database consists of mostly privately insured individuals, so the findings may be generalized to the broader population of women with commercial insurance in the U.S.

Some of the limitations of this study are as follows. As with any claims-based surveillance of prescription drug use, this study assumed that prescriptions dispensed were taken by the women. Exposure was defined by the days supplied from each dispensing. Additionally, pregnancies were counted in every drug category to which they were exposed, but this study did not determine how many pregnancies were exposed to multiple medications, or the reason for changes in treatment. Furthermore, the results are limited to the pregnancies with a livebirth outcome, and do not include exposures in women who had miscarriages, stillbirths, or terminations. At the time of this study, we were unable to define women’s parity. Out-of-pocket payments for prescriptions are not recorded in claims data and are a source of missing data. The definition for pre-gestational diabetes is not validated, and misclassification of diabetes status is possible. Finally, data partners contributed data for different time periods, with most contributing data for recent years, so these estimates most closely reflect use in the 2007–2013 time period. This limitation means we could not analyze trends robustly over time, although some general observations are similar to the trends others have described in similar populations [17, 18]. Among all livebirth pregnancies in our study, the use of glyburide increased over this time period studied, from less than 0.5% of pregnancies to approximately 2%; this increase was not seen in pregnancies among women with pre-existing diabetes (data not shown). For women with pre-existing diabetes, the prevalence of metformin use has increased (20.4 to 41.5%) over the study period, while use of insulin decreased (89.8 to 77.3%). Interpreting these trends requires the assumption that the women enrolled in the databases contributing data in the early years are similar to the women enrolled in the contributing databases in recent years, which may not be a valid assumption.

The rates of diabetes mellitus and gestational diabetes have increased over the last two decades [1, 2, 4, 6, 19]. Obstetric practice recommendations in the treatment of gestational diabetes has also changed, as noted by others and as reflected in updated guidelines [8, 11, 18]. These trends suggest that a larger proportion of pregnancies are likely exposed to antidiabetic drugs in the coming years. Changes in disease incidence and management highlight the need for continuing surveillance of ADD utilization patterns and examining the safety and effectiveness of these products in pregnancy.

## Conclusions

This study finds that from 2001 to 2013, 4.4% of pregnancies were exposed to an antidiabetic drug. Almost all pregnancies among women with pre-existing diabetes continue treatment during the pregnancy, with 75% being treated with insulin. Among pregnancies in women without pre-existing diabetes, sulfonylureas (particularly glyburide) and insulin were the most commonly used products to treat gestational diabetes. This observational cohort of pregnancies receiving ADD treatment highlight the importance of continuing surveillance of ADD utilization patterns and examining drug safety and effectiveness among pregnant women.

## Data Availability

The datasets used and/or analyzed during the current study are available from the corresponding author on reasonable request.

## Abbreviations

ACIP: Advisory Committee on Immunization Practices
ACOG: American College of Obstetricians and Gynecologists
ADD: Antidiabetic drug
FDA: Food and Drug Administration
ICD-9: International Classification of Diseases, Ninth Edition
LMP: Last menstrual period
MEPREP: Medication Use in Pregnancy Risk Evaluation Program
MSDD: Mini-Sentinel Distributed Database
PR: Prevalence ratio
PRISM: Post-Licensure Rapid Immunization Safety Monitoring
